# Revision of the endemic Taiwanese millipede genus *Aponedyopus* Verhoeff, 1939, with descriptions of two new species (Diplopoda, Polydesmida, Paradoxosomatidae). Advances in the systematica of Diplopoda III

**DOI:** 10.3897/zookeys.72.743

**Published:** 2010-12-17

**Authors:** Chao-Chun Chen, Sergei I. Golovatch, Hsueh-Wen Chang

**Affiliations:** 1 Department of Biological Sciences, National Sun Yat-Sen University, 70 Lien-hai Rd. Kaohsiung, Taiwan 804, ROC; 2 Institute for Problems of Ecology and Evolution, Russian Academy of Sciences, Leninsky pr. 33, Moscow 119071, Russia

**Keywords:** Millipede, *Aponedyopus*, taxonomy, new species, distribution, key, Taiwan

## Abstract

The millipede genus Aponedyopus is endemic to Taiwan and contains three species*.* All previously described nominal species are considered to represent one species: Aponedyopus montanus Verhoeff, 1939 (the type species), including Aponedyopus reesi (Wang, 1957) and Aponedyopus maculatus Takakuwa, 1942, **syn. n.** Two further species are described as new: Aponedyopus similis **sp. n.** and Aponedyopus latilobatus **sp. n.** The genus is re-diagnosed, all of its three species are keyed, and their distributions mapped.

## Introduction

The genus Aponedyopus Verhoeff, 1939 was first proposed to incorporate the single species Aponedyopus montanus Verhoeff, 1939 said to be from the foot of Mt Fuji in Japan. The generic diagnosis was very poor, relying on a highly superficial resemblance in the gonopod conformation of Aponedyopus to the genus Nedyopus Attems, 1914 (Verhoeff 1939). Takakuwa (1942) was the first to add another species to this genus, Aponedyopus maculatus Takakuwa, 1942 from Taiwan, and, later (1954), questioned the provenance of Aponedyopus montanus from Honshu, suggesting it had also derived from Taiwan. (Wang (1957a, 1957b) described two further species from Taiwan which he first placed in Nedyopus: Nedyopus reesi Wang, 1957 and Nedyopus jeanae Wang, 1957; then (1963a, 1963b) he transferred the latter species to Aponedyopus and referred to it as only a subspecies of Aponedyopus montanus. Finally, in a checklist of the Taiwanese Diplopoda, Wang (1964) listed the following Aponedyopus: Aponedyopus montanus montanus, Aponedyopus montanus jeanae, Aponedyopus reesi, and Aponedyopus maculatus, the former subspecies quoted as stemming from Japan and being common there, as opposed to the latter three taxa which were said to be endemic to Taiwan. Thus, Wang ignored the previous doubts expressed by Takakuwa (1954) concerning the origin of Aponedyopus montanus in Japan. He also neglected Miyosi (1959) who had formally synonymized Aponedyopus jeanae with Aponedyopus montanus and who had also agreed with Takakuwa that Aponedyopus montanus had to stem from Taiwan, not from Japan. Hoffman (1980) accepted that the genus occurred in both Taiwan and Japan. Murakami (1993) included Aponedyopus montanus in the most recent checklist of the Japanese millipedes, but Shinohara and Tanabe (1999) emphasized that the original, type locality of Aponedyopus montanus, i.e. Mt Fuji, might have been mislabeled. Yet, the genus Aponedyopus still remains on the generic list of Japanese Myriapoda (Tanabe 2001).

Jeekel (1968) was the first to properly, however succinctly, re-diagnose Aponedyopus, emphasizing it had nothing to do with the stem-name Nedyopus, because these genera show vastly different courses of their seminal grooves and several other important details of gonopod structure, and belong in different tribes. Yet Jeekel mistakenly listed Aponedyopus jeanae as a valid species and erroneously believed he was the first to transfer both Aponedyopus jeanae and Aponedyopus reesi to Aponedyopus. In fact, Miyosi (1959) had done it before, followed also by Wang (1964).

Korsós (2004), in the latest catalogue of the Diplopoda of Taiwan, listed only two species in Aponedyopus: Aponedyopus montanus and Aponedyopus maculatus. Concerning the former species, he listed two junior synonyms claimed as new: Aponedyopus reesi and Aponedyopus montanus jeanae. However, he must have overlooked Miyosi (1959), who had already synonymized the latter taxon under Aponedyopus montanus. He also erred in stating that Aponedyopus maculatus had been described from Ikao, Japan, whereas it had actually been described from Piyanan (= Sihyuanyakou (思源啞口), Datong Township(大同鄉), Yilan County (宜蘭縣)), Taiwan (Takakuwa 1942). In addition, not only all of the previous records of these species in Taiwan were summarized, but he also provided some new localities for Aponedyopus maculatus.

The present study reviews the millipede genus Aponedyopus, based on abundant fresh material, including some near-topotypes of one of included species, covering various parts of Taiwan. Thus, the previously described species could be re-assessed, two new species added, and anew synonym established.

## Material and methods

New extensive collections of millipedes covering most parts of Taiwan were made between 1989 and 2009, using hand-sorting of the soil and litter. Specimens were preserved in 70% ethanol. External structures were examined and the drawings prepared with a LEICA MZ 16 stereomicroscope, as well as with a HITACHI S2400 scanning electron microscope. Coloration of the specimens is described from alcohol material. This material has been shared between the collections of Department of Life Science, National Chung Hsing University (NCHUL), Taiwan; Department of Biological Sciences, National Sun Yat-Sen University (NSYSUB), Taiwan; Department of Life Sciences, National Taiwan Normal University (NTNUL), Taiwan; National Museum of Natural Science (NMNS), Taiwan; Taiwan Forestry Research Institute (TFRI), Taiwan; and Zoological Museum of the State University of Moscow (ZMUM), Russia.

## Systematic Account

### Genus Aponedyopus

Verhoeff, 1939

Aponedyopus Verhoeff 1939: 119; Takakuwa 1954: 49; Jeekel 1968: 75; Hoffman 1980: 170; Shinohara and Tanabe 1999: 681.

#### Diagnosis.

Medium- to large-sized Paradoxosomatidae (15–55 mm long, 2.0–5.0 mm wide) with 20 segments. Pore formula normal. Paraterga poorly developed, evident only on segment 2. An evident sternal lobe between ♂ coxae 4; ♂ segment 7 with or without a pair of prominent sternal cones (= spiracles) flanking gonopod aperture. ♂ tarsal brushes present.

Gonopod coxae long, subcylindrical, setose distodorsally, cannula as usual. Telopodites rather long, their distal parts crossingmedially *in situ*. Femorite long, moderately to evidently broadened parabasally on dorsal side, apically separated from postfemoral region by a clear oblique sulcus on lateral side; postfemoral part enlarged at base, tapering thereafter, demarcated from solenophore by a sulcus on mesal side; solenophoreshorter than to as long as femorite, curved first ventrad and then dorsad on mesal face, distally holding subparallel to broadened part of femorite; base of solenophore with a small to obvious, apically deeply bifid lobe; seminal groove first running fully on mesal face of femorite, then turning dorsad near postfemoral part and continuing onto solenomere at base of solenophore on dorsal face; solenomereflagelliform, long, at most only slightly longer than, and nearly completely supported/sheathed by, solenophore, with only tip of solenomere sometimes exposed.

### Aponedyopus montanus

Verhoeff, 1939

**Figures 1–4.**
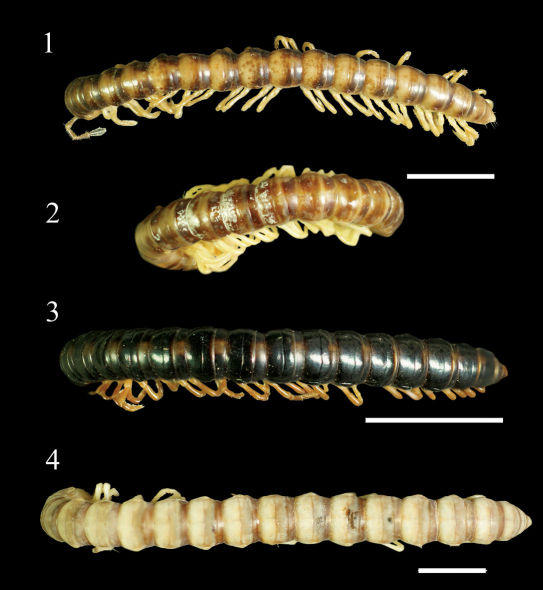
Aponedyopus montanus Verhoeff, 1939, showing different colour patterns, ♂♂ from Mt Taiping (太平山) **1** Zjhong (自忠) **2** Yima forest path (依麻林道)**3** NanSi forest path (楠溪林道) **4** dorsal view. Scale bars: 5.0 mm.

Figs 1 [Fig F2] [Fig F3] [Fig F4] 23 40–43 50–53

Aponedyopus montanus Verhoeff 1939: 119–121, figs 5–7.
Aponedyopus montanus Miyosi 1959: 73; Jeekel 1968, 75; Wang and Mauriès 1996: 87; Korsós 2004: 21.
Aponedyopus montanusmontanus Wang 1964, 69.
Aponedyopus maculatus Takakuwa 1942: 238, figs 3 & 4, syn. n.
Aponedyopus maculatus Wang 1958: 342; 1963a: 90; 1964: 69; Jeekel 1968: 75; Wang and Mauriès 1996: 87; Korsós 2004: 20.Nedyopus reesiWang 1957a: 104–106, fig. 2; first synonymized by Korsos 2004.
Nedyopus reesi Wang 1958: 342.
Aponedyopus reesi Miyosi 1959: 73; Wang 1964: 69; Jeekel 1968: 75; Wang and Mauriès 1996: 87.
Nedyopus jeanae Wang 1957b: 113–115, fig. 8; first synonymized by Miyosi 1959.
Nedyopus jeanae Wang 1958: 342; Miyosi 1959: 73.
Aponedyopus jeanae Wang 1963b: 288; Jeekel 1968: 75; Wang and Mauriès 1996: 87.
Aponedyopus montanus jeanae Wang 1963a: 90; 1964: 69.

#### Material examined:

1 ♀ (NSYSUB-DI 60), Taiwan, Taipei City, BeiTou area (北投區), 101 Jiia county road (101甲縣道), ca 860 m a.s.l., 4 May 2002, leg. S. Y. Wu. 1 ♂ (NCHUL), Taipei County (台北縣), Gongliao Township (貢寮鄉), upstream of Yuanwangkeng Stream (遠望坑溪上游), 6 June, 1998, leg. S. H. Wu. 1 ♂ (NSYSUB-DI 67), Taipei County (台北縣), ULai Township (烏來鄉), TaManShan (塔曼山), in decaying wood, 2,100 m a.s.l., 23 August 2002, same collector. 1 ♀ (NSYSUB-DI 59), same township, WuLai (烏來), 1,000–1,200 m a.s.l., March 2002, leg. C. C. Chen & C. S. Iang. 1 ♂, 2 ♀ (TFRI), same township, FuShan Botanical garden (福山植物園), ca 730 m a.s.l., 18–25 May 2001, leg. W. B. Huang. 1 ♂, 3 ♀ (NSYSUB-DI 61–64), Taiwan, Taoyuan County (桃園縣), FuSiing Township (復興鄉), HuaLeng Village (華稜村), Northern Cross-Island Highway (北部橫貫公路)/Provincial # 7 Highway (台七線), 53 km, ca 1,030 m a.s.l., 22 April 2003, same collector. 1 ♀ (NSYSUB-DI 73), same locality, 58 km, ca 1,110 m a.s.l., 29 May 2003, same collector. 1 ♀ (NSYSUB), same locality, 56 km, ca 1,030 m a.s.l., 23 June 2006, same collector. 3 ♂ (NSYSUB-DI 444–446), same township, Baling (巴陵), ca 600 m a.s.l., 3 April 2004, leg. H. D. Zhu. 1 ♀ (NTNUL-My 15), Hsinchu County (新竹縣), Wufeng Township (五峰鄉), GuanU (觀霧), ca 2,000 m a.s.l., 28 June 1993, leg. S. H. Chen. 1 ♂ 1 ♀ (NSYSUB), same township, ShihLu old path (石鹿古道), ca 1,600 m a.s.l., 22 September 2005, leg. H. D. Zhu. 1 ♀ (NSYSUB), same township, Syueba farm (雪壩農場), DaLu forest path (大鹿林道), ca 1,890 m a.s.l., 1 October 2006, leg. S. Y. Wu. 1 ♂, 1 ♀ (ZMUM), Yilan County (宜蘭縣), Yuanshan Township (員山鄉), Shuanglian Pond (雙連埤), ca 500 m a.s.l., 11 May 2007, same collector. 1 ♀ (NSYSUB), Datong Township (大同鄉), Cueifong Lake (翠峰湖), ca 1,900 m a.s.l., 29 July 2004, same collector. 1 ♂ (NSYSUB), same township, Northern Cross-Island Highway (北部橫貫公路)/Provincial # 7 Highway (台七線), MingChih (明池), ca 1,200 m a.s.l., 13 April 2006, same collector. 1 ♂ (NSYSUB), same township, Mt Taiping (太平山), ca 1,930 m a.s.l., 26 February 2007, 24°28'46"N, 119°31'03"E, leg. M. H. Hsu. 1 ♀ (NSYSUB), same township, forest path # 100 (100號林道), 21 km, ca 1,600 m a.s.l., 9 September 2009, leg. C. J. Jheng. 1 ♂ (NSYSUB-DI 65.), Taichung County (台中縣), HePing Township (和平鄉), AnMaShan forest amusement zone (鞍馬山森林遊樂園), ca 2,000 m a.s.l., 7 May 2003, leg. S. Y. Wu. 1 ♂ (NCHUL), Nantou County (南投縣), LuGu (鹿谷鄉), SiTou (溪頭), ca 1,140 m a.s.l., 31 October 1997, leg. S. H. Wu. 1 ♀ (NCHUL), same locality, ca 1,160–1,400 m a.s.l., 31 October 1997, leg. S. H. Chen. 1 ♀ (NSYSUB-DI 58), same locality, ShenMu walking path (神木步道), under stones, ca 1,200 m a.s.l., 15 November 2002, leg. J. D. Lee. 1 ♀ (JDLee20021114008, deposited at NSYSUB), same locality, TuDiGongLun walking path (土地公崙步道), ca 1,160–1,400 m a.s.l., same date and collector. 2 juveniles (JDLee20021114004, deposited at NSYSUB), same locality, SiTou walking path (溪頭步道), same date and collector. 3 ♀ (NTNUL-My 6–9), same county, ShinYi Township (信義鄉), Zjhong (自忠), ca 2,340 m a.s.l., 1 July 1989, leg. S. H. Chen. 3 ♂ (NTNUL-My 25–28), same locality, date and collector. 1 ♀ (NSYSUB), same county, Zhushan Township (竹山鎮), ShanLinSi amusement park (杉林溪遊樂園), ca 1,600 m a.s.l., 7 October 2004, leg. S. Y. Wu. 1 ♂ (NSYSUB), Taiwan, Hualien County (花蓮縣), Xiulin Township (秀林鄉), Mt. JiaLiWan (加禮宛山), ca 1,290 m a.s.l., 29 July 2005, leg. F. S. Jhou. 1 ♀ (NSYSUB), same township, Toroko (太魯閣), Lianhua Pond walking path (蓮花池步道), ca 1,060 m a.s.l., 24°13'10"N, 119°28'49"E, 28 February 2007, leg. M. H. Hsu. 1 ♀ (NSYSUB), same county, FengBin Township (豐濱鄉), Ruigang Highway (瑞港公路), ca 130 m a.s.l., 23°28'50"N, 119°27'31"E, 7 May 2009, leg. M. H. Hsu. 2 ♂ (NSYSUB-DI 69–70), same county, JhuoSii (卓溪鄉), WaLaMi (瓦拉米), YuShan National Park (玉山國家公園), ca 1,080 m a.s.l., 24 February 2003, leg. H. D. Zhu. 1 ♂, 3 juveniles (NTNUL-My 29–32), Chia-I County (嘉義縣), ALiShan Township (阿里山鄉), ALiShan (阿里山), ca 2,260 m a.s.l., 11 March 1989, leg. S. H. Chen. 1 ♂, 2 ♀ (NTNUL-My 49–51), 2,250 m a.s.l., 3 July 1989, same locality and collector. 1 ♀ (NSYSUB-DI 74), same locality, ALiShan amusement park (阿里山遊樂園), under stones on soil, ca 2,280 m a.s.l., 24 June 2003, leg. Y. H. Lin. 1 ♀ (NSYSUB-DI 66), Kaohsiung County (高雄縣), TaoYuan (桃源鄉), TengJhih (藤枝), ShihShan forest path (石山林道), 6 km, ca 1,600 m a.s.l., 21 August 1998, collector unknown. 1 ♂ (NSYSUB-DI 66), same locality, 1 August 2001, leg. C. R. Wu. 1 ♀ (NSYSUB-DI 72), same locality, ca 1,450 m a.s.l., 14 April 2003, leg. S. Y. Wu. 1 ♂ (NSYSUB), same township, NanSi forest path (楠溪林道), ca 2,000 m a.s.l., 24 September 2002, leg. M. J. Hong & M. J. Wu. 1 ♀ (NSYSUB), same township, Southern Cross-Island Highway (南部橫貫公路), DaGuanShan (大關山), YaKou forest path (啞口林道), ca 2,720 m a.s.l., 13 May 2007, leg. Y. C. Chang. 1 ♀ (NSYSUB-DI 71), at boundary between MaoLin County (茂林鄉) of Kaohsiung and UTai County (霧臺鄉) of PingTung, YuGuTing (雨谷亭), under stone, ca 2,150 m a.s.l., 28 March 2003, leg. H. W. Chang. 1 ♂ (NSYSUB), Taitung County (台東縣), JinFeng Township (金峰鄉), Yima forest path (依麻林道), ca 1,110 m a.s.l., 2 July 2009, leg. M. H. Hsu. 1 ♀ (NSYSUB), PingTung County (屏東縣), ChunRih Township (春日鄉), DaHan forest path (大漢林道), 20 km, under stone, ca 250 m a.s.l., 9 July 2004, leg. W. J. Lee. 2 ♂ (NSYSUB), same county, Taiwu Township (泰武鄉), entrance to North DaWu Mountain (北大武山登山口), ca 1,400 m a.s.l., 23 January 2004, leg. H. D. Zhu.

**Figures 5–7. F2:**
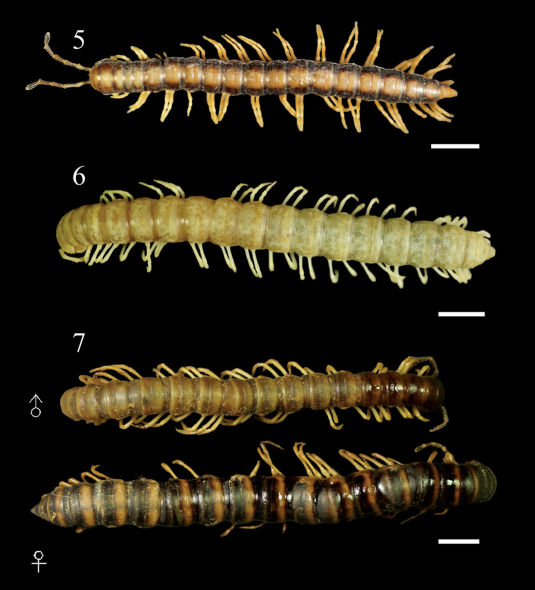
Aponedyopus montanus Verhoeff, 1939, showing different colour patterns, ♂♂ from upstream of Yuanwangkeng Stream (遠望坑溪上游) **5** ShanLinSi amusement park (杉林溪遊樂園) **6** ShihLu old path (石鹿古道) **7** dorsal view. Scale bars: 5.0 mm.

**Figures 8–11. F3:**
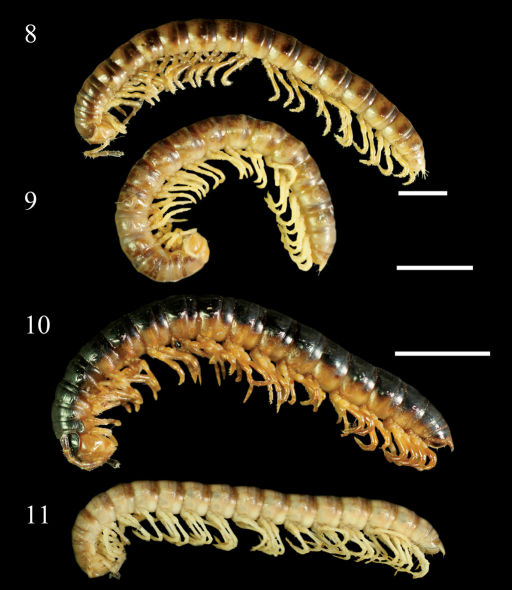
Aponedyopus montanus Verhoeff, 1939, showing different colour patterns, ♂♂ from Mt Taiping (太平山) **8** Zjhong (自忠) **9** Yima forest path (依麻林道) **10** NanSi forest path (楠溪林道) **11** lateral view. Scale bars: 5.0 mm.

**Figures 12–14. F4:**
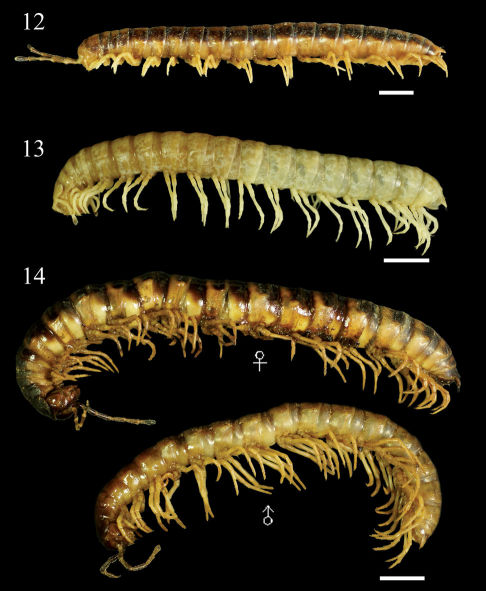
Aponedyopus montanus Verhoeff, 1939, showing different colour patterns, ♂♂ from upstream of Yuanwangkeng Stream (遠望坑溪上游) **12** ShanLinSi amusement park (杉林溪遊樂園) **13** ShihLu old path (石鹿古道) **14** dorsal view. Scale bars: 5 mm.

**Figure:**
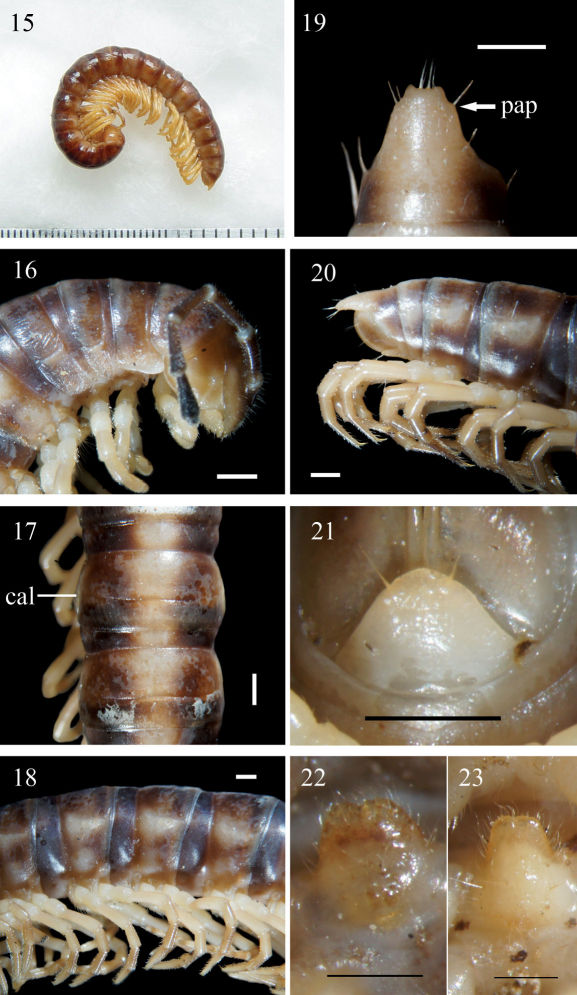
Aponedyopus montanus Verhoeff, 1939, ♂ from Mt JiaLiWan (加禮宛山). **15** Entire body, lateral view **16** Anterior body portion, lateral view. **17, 18** Midbody segments, dorsal and lateral views, respectively **19–20** Epiproct (**epi**), dorsal and lateral views, respectively **21** Hypoproct (**hyp**), ventral view **22, 23** Sternal lobe between ♂ coxae 4, subventral views. Scale bars: 1.0 mm for 15–21, 0.5 mm for 22, 23. **al:** axial line; **cal:** calluses; **col:** collum; **meta:** metazona; **o:** ozopore; **pap:** pre-apical papillae; **par:** paraterga; **ple:** pleurosternal region; **pro:** prozona; **rug:** rugulose; **str:** stricture; **su**l: transverse sulcus; **tar:** tarsal brushes.

#### Diagnosis:

Differs from the other Aponedyopus species in often containing specimens considerably more than 40 mm long, in the considerably longer ♂ legs (usually about twice as long as midbody height), a dentiform process **b** at the base of the gonopod prefemoral part and, above all, the slender terminal branches (**x** and **y**) of the solenophore (Figs 40, 42 & 43).

#### Description:

Length 40–55 (♂, n=11) or 47–58 mm (♀, n = 13); width of midbody metazona 10 ca 3.5–5.0 (♂) or 5.0–6.0 mm (♀).

Coloration in alcohol entirely light yellow to dark brown (Figs 1–14). Antennae light yellow to dark brown, increasingly blackish distally, but tip pallid; head to anterior half of epiproct (**epi**) (Fig. 19), pleurosternal region (**ple**) (Fig. 18) light yellow to dark brown, prozona (**pro**) always darker than metazona (**meta**) (Fig. 18), anterior and hind edges of metazona evidently to slightly lighter brown; posterior half of epiproct, sterna and legs light yellow to orange-brown in ♂.

Head densely setose in clypeolabral region, vertex nearly bare, epicranial suture distinct. Postcollum constriction faint; in width, segments 2 = 3 = 4 < head = segment 5 = 6 < collum (**col**) (Fig. 16) = segments 7–17 in ♂, or segments 2 = 3 = 4 < head < collum = segments 5–16 in ♀; thereafter body gradually and gently tapering both in width and height towards telson. Antennae medium-sized to long, stout, reaching behind middle of metatergite 3 to middle of metatergite 4 dorsally (♂) (Fig. 16), or midway to end of segment 3 (♀). Surface generally shining and rather smooth, only metaterga rugulose (**rug**) (Fig. 16) (post-sulcus halves (Fig. 17) usually slightly more so); surface below paraterga (**par**) (Fig. 18) visibly and densely granular on anterior segments, increasingly sparsely granular towards telson in both sexes, sometimes densely granular until segment 19 in ♀. Paraterga (**par**) (Fig. 18) poorly developed, especially evident as low ridges drawn considerably forward into a rounded lobe on segment 2 in both sexes, nearly to totally wanting on segments 16–19 (sometimes only a dorsal sulcus above ozopore (**o**) (Fig. 18) still present); calluses (**cal**) (Fig. 17) always delimited by a sulcus dorsally, calluses thinner on poreless segments, broader on pore-bearing ones, but a ventral sulcus mostly observed in caudal 1/3 only until segment 15; paraterga even more strongly reduced in ♀. Axial line (**al**) usually absent to traceable in places on collum and following metaterga, sometimes evident on metaterga in both sexes (Fig. 17). A medially sinuate transverse sulcus (**sul**) (Fig. 17) evident on segments 5–17, traceable on segments 4 and 18(19) in both sexes, narrow, shallow, very faintly beaded to smooth at bottom, not reaching bases of paraterga. Limbus (= region between two arrows, Fig. 16) thin, caudal margin entire. Stricture (**str**) (Fig. 17) between pro- and metazona shallow, narrow, faintly ribbed at bottom in both sexes. Pleurosternal carinae (arrow) (Fig. 16) nearly wanting, present as slight flaps only on segment 2, barely traceable on segment 3 (Fig. 16). Tergal setae almost fully abraded, pattern traceable mostly as 1+1 or 2+2 insertion points at anterior edge of collum in both sexes, as well as 2+2 in anterior (pre-sulcus) and 2+2 in posterior (post-sulcus) row on following metaterga. Ozopores (**o**) (Fig. 18) lateral, lying on callus ca 1/3 metatergal length in front of caudal edge (Figs 17 & 18). Epiproct (**epi**) (Figs 19 & 20) moderately long, conical, only slightly curved in lateral view, ratio of epiproct length to pre-epiproct length of telson 1.3:1 in ♂, tip emarginated in both sexes in dorsal view (Fig. 19); pre-apical papillae (**pap**) (Fig. 19) evident, close to apex. Hypoproct (**hyp**) (Fig. 21) usually subtrapeziform (♂, ♀), more rarely subtriangular to semi-circular (♀), 1+1 setae at caudal corners situated on well-separated knobs, sides straight (♂) or slightly convex (♀).

Sterna sparsely setose, each cross-impression with neither a transverse sulcus nor an axial groove; a slightly to very slightly notched, setose, ventrally bulging lamina only between ♂ coxae 4 (Figs 22 & 23). Ridges/cones (= spiracles) flanking gonopod aperture present or absent. Legs long, ca twice as long as midbody height, shorter and slenderer in ♀; legs 1 to posterior legs of segment 15 with obvious tarsal brushes (**tar**) (Fig. 18) only in ♂, ♀ without tarsal brushes; ♂ coxa 2 with a small apical process carrying a gonopore.

Gonopods (Figs 40–43, 50–53) simple. Coxite (**cx**) (Fig. 41) elongate, subcylindrical, setose distodorsally; cannula normal. Telopodites (**T**) (Fig. 40) curved distally, longer than coxite. Prefemoral part (**pf**) (Fig. 41) short and stout, almost 1/3 femur length, as usual densely setose. Femorite (**fe**) (Fig. 41) evidently broadened near base on dorsal side, with a clear demarcation sulcus (**su**) (Fig. 41) on lateral side separating a postfemoral part **(pst)** (Fig. 41); the latter showing an obvious, spiniform, (nearly) pointed branch (**b**) (Figs 42 & 43) parabasally on lateral side; solenophore (**sph**) (Fig. 41) with another demarcation sulcus separating it from **pst** on medial side, long, only slightly shorter than to as long as femorite, twisted and curved first ventrad and then dorsad on medial side in ventral view, distally holding subparallel to broadened part of femorite; base of **sph** with an obvious, subspiniform lobe (**l**) (Fig. 40), either well separated from or holding quite adjacent to **sph** base; terminal part of **sph** divided into two slender, separated branches: one wide, flattened dorsoventrally, with a rounded membranous end (**y**), the other spiniform (**x**) (Fig. 40). Seminal groove (**sg**) (Fig. 50) first running fully on mesal face of **fe**, then turning dorsad near **pst** to continue onto solenomere (**sl**) (Fig. 41) at base of **sph** on dorsal face; **sl** flagelliform, long, only slightly longer than **sph** and nearly completely supported/sheathed by **sph**, only tip of **sl** exposed.

#### Distribution:

Type material has not been revised, presumably in the collection of the Zoologische Staatssammlung in Munich, Germany.

This species is highly variable in size and coloration, and is the most widespread amongst Aponedyopus species in Taiwan. Its distribution covers much of the island and vertically ranges from 175 to over 2,720 m a.s.l. (Map).

### Aponedyopus similis sp. n.

urn:lsid:zoobank.org:act:80CFF96F-2331-45EB-B4B5-DF5814A57660

Figs 24–31 44, 45 54 & 55 

#### Material examined:

Holotype ♂ (TFRI), Taiwan (R. O. C.), Taichung County (台中縣), HePing (和平鄉), Shengguang (勝光), ca 2,200 m a.s.l., 26 March – 25 April, 2003, leg. W. C. Yeh.

Paratype ♂ (NSYSUB-DI 75), Taiwan (R. O. C.), Hsinchu County (新竹縣), Wufeng Township (五峰鄉), GuanU (觀霧), 24.5 km from entrance to national park, ca 2,000 m a.s.l., 13 August 2002, leg. C. C. Chen, Y. H. Lin & J. N. Huang.

**Figures 24–31.**
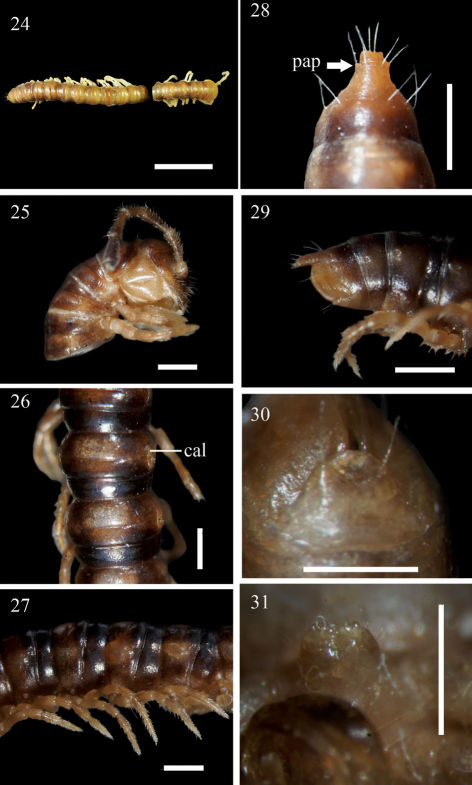
Aponedyopus similis sp. n., ♂ holotype (24) ♂ paratype (25–31). **24** Entire body, dorsal view **25** Anterior body portion, lateral view **26–27** Midbody segments, dorsal and lateral views, respectively **28–29** Epiproct, dorsal and lateral views, respectively **30** Hypoproct, ventral view **31** Sternal cones near gonopod aperture. Scale bars: 1.0 mm for 24–29, 0.5 mm for 30, 31. **cal:** calluses; **pap:** pre-apical papillae.

#### Name:

To emphasize the close resemblance to the next new species.

#### Diagnosis:

Being apparently the most similar to Aponedyopus latilobatus sp. n., based both on several peripheral characters (shorter legs, mostly a smaller body size etc.) and gonopod conformation, it is distinguished by the gonopod lobe **b** being membranous and lobiform, the terminal branches of the solenophore differing in length and crossing each other, with branch **x** carrying an inconspicuous lobe (see also Key below).

#### Description:

Length ca 22 mm (♂, n=2); width of pro- and metazona 10 ca 1.8 and 2.0 mm, respectively.

General coloration in alcohol brown to dark brown (Figs 24–27), with a clear pattern of a lighter brown to yellow brown axial stripe consisting of narrower subtriangular spots on proterga and twice as wide central spots on metaterga, these spots growing slightly infuscate, to blackish both towards stricture and posterior half of metaterga; prozona slightly darker than metazona, thus providing a vague cingulate pattern as well; paraterga, legs and venter slightly lighter than background, light grey-brown; head marbled brown, especially well so in vertigial region, genae contrastingly yellowish, a square median spot above antennal sockets contrastingly dark brown; antennae increasingly infuscate, up to blackish distad, distinctly darker at margins, marbled and lighter centrally, only tip contrastingly pallid; both collum and segment 2 with a very faint, yellow-brown, axial line; epiproct uniformly light brown, only very slightly infuscate near base.

Postcollum constriction evident; in width, segment 2 = 3 < 4 < collum < head = segments 5–15; thereafter body gradually and gently tapering towards telson both in width and height. Antennae (Fig. 25) medium-sized, slender, reaching behind stricture of tergite 3. Paraterga (Figs 26 & 27) very poorly developed, very evident and low only on segment 2, calluses (**cal**) (nearly) completely delimited by a sulcus dorsally, in caudal 1/3 also ventrally only on pore-bearing segments. Transverse sulcus (Figs 26, 27) developed on segments 5–17, traceable on segment 18, wanting on 19^th^, narrow, shallow, neither beaded at bottom nor reaching bases of paraterga. Surface smooth throughout, slightly granulated only below paraterga 2–4. Limbus thin, caudal margin entire. Stricture dividing pro- and metazona shallow, narrow, not beaded at bottom (Figs 26 & 27). Pleurosternal carinae present only on segments 2 and 3 (Fig. 25). Tergal setae almost fully abraded, 2+1 retained only at anterior edge of collum; pattern untraceable. Ozopores lateral, lying on calluses ca 1/2 metatergal length in front of caudal edge (Figs 26 & 27). Epiproct long (Figs 28 & 29), flattened dorsoventrally, straight, not curved caudoventrad in lateral view, ratio of epiproct length to pre-epiproct length of telson 1: 1.3, tip of epiproct slightly concave; pre-apical papillae (**pap**) evident, close to apex. Hypoproct (Fig. 30) rounded, subtrapeziform, 1+1 setae at caudal corners situated on well-separated knobs, sides slightly concave.

Sterna sparsely setose; lamina between coxae 4 setose and emarginate (Fig. 31); segment 7 with a pair of prominent sternal cones (= spiracles) flanking gonopod aperture; each cross-impression with a transverse sulcus, but without axial groove. Legs (Fig. 27) moderately long and slender, legs 1 to anterior legs of segment 17 with tarsal brushes, thereafter legs broken off in both available ♂♂, each midbody leg ca 1.2 times as long as body height, coxa 2 with a small apical process supporting a gonopore.

Gonopods (Figs 44, 45, 54 & 55) with process **b** at base of postfemoral part lobe-shaped, membranous, not like a distinct process; **l** at base of solenophore rather vague; distal part of gonopod deeply bifid, divided into a longer solenomere (**sl**), more complex at end and bearing a low terminal lobe, and a slightly shorter, simple, nearly pointed solenophore branch (**sph**); ends of both branches crossing.

#### Distribution:

This species seems to be local, occurring only rather high (2,000–2,200 m a.s.l.) in the mountains of northern Taiwan (Map).

### Aponedyopus latilobatus sp. n.

urn:lsid:zoobank.org:act:9365404E-6E43-4CBA-ABA9-4C0466D27E42

Figs 32–39 46–49 56–58 

#### Material examined:

Holotype ♂ (NSYSUB-DI 76), Taiwan (R. O. C.), Taichung County (台中縣), HePing (和平鄉), Sihyuanyakou (思源啞口), forest path no. 710, 1.5 km from entrance to path, ca 2,050–2,100 m a.s.l., 21 August 2002, leg. C. C. Chen & Y. H. Lin.

Paratypes: 3 ♀ (NSYSUB-DI 77–79), same locality, date, and collectors, together with holotype.

**Figures 32–39.**
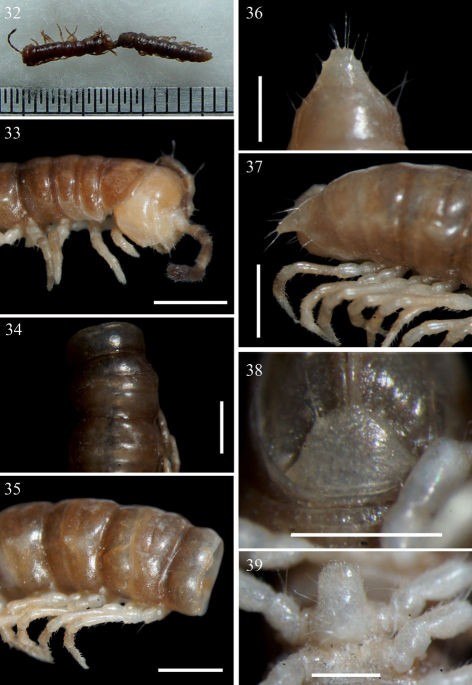
Aponedyopus latilobatus sp. n., ♂ holotype. **32** Entire body, dorsal view **33** Anterior body portion, lateral view **34–35** Midbody segments, dorsal and lateral views, respectively **36–37** Epiproct, dorsal and lateral views, respectively **38** Hypoproct, ventral view **39** Sternal lobe between coxae 4. Scale bars: 0.5 mm for 36, 39, 1.0 mm for others.

**Figures 40–43.**
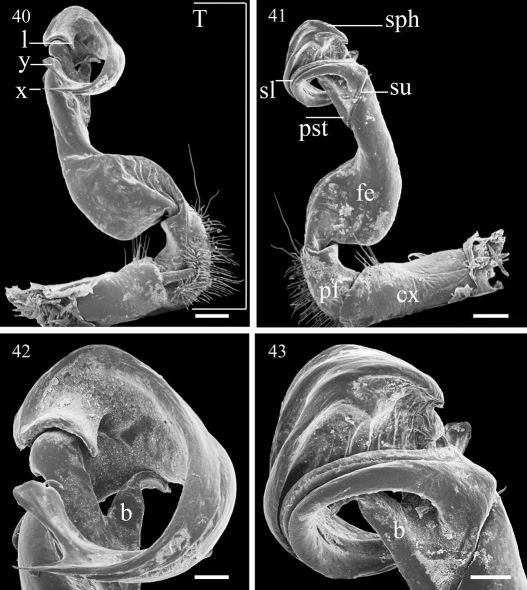
Aponedyopus montanus Verhoeff, 1939, ♂ fromFuShan Botanical Garden (福山植物園), left gonopod, (40, 41) mesal and lateral views, respectively **42–43** telopodite tip, mesal and lateral views, respectively. Scale bar = 0.5 mm for 40, 41, 0.2 mm for 42, 43. **cx**: coxite, **b**: spiniform pointed branch, **fe**: femorite, **l**: lobe, **pf**: prefemoral part, **pst**: postfemoral part, **sl**: solenomere, **sph**: solenophore, **su**: sulcus, **T**: telopodite.

**Figures 44, 45.**
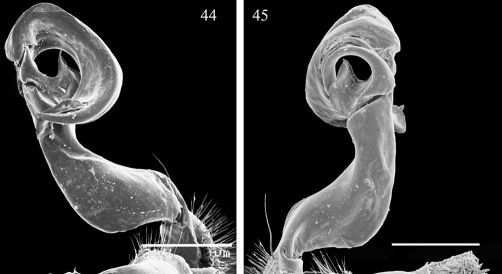
Aponedyopus similis sp. n., ♂ paratype, left gonopod **44, 45**, submesal and sublateral views, respectively. Scale bar = 0.5 mm.

**Figures 46–49.**
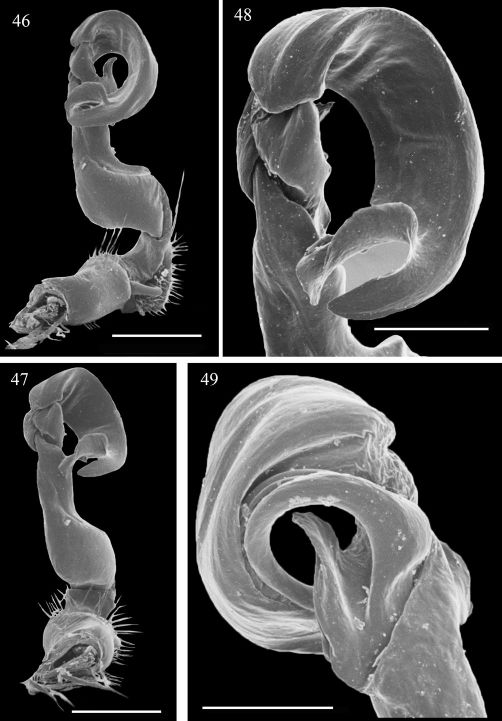
Aponedyopus latilobatus sp. n., ♂ holotype, left gonopod **46, 47**, submesal and dorsal views, respectively **48, 49** telopodite tip, submesal and lateral views, respectively. Scale bar = 0.5 mm for 46, 47, 0.25 mm for 48, 49.

**Figures 50–53.**
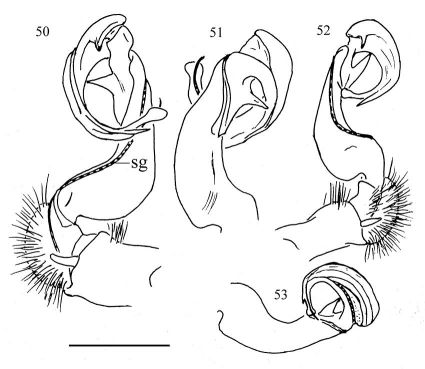
Aponedyopus montanus Verhoeff, 1939, ♂♂. **50, 51** right gonopod, mesal and lateral view, respectively. **52–53** left gonopod, mesal and lateral view respectively. Scale bar = 1 mm. **sg**: seminal groove.

**Figures 54–58.**
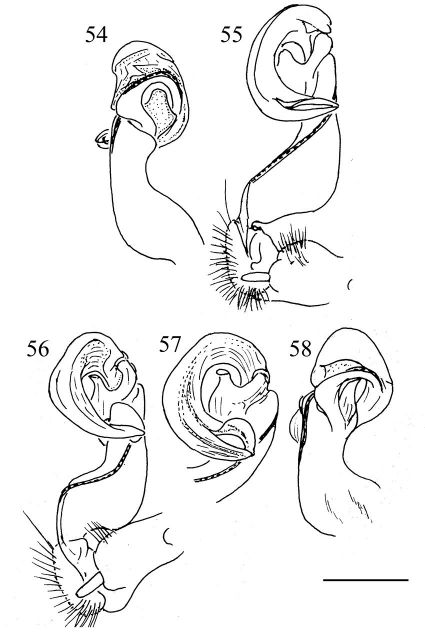
Aponedyopus similis sp. n., ♂ paratype, right gonopod, lateral and mesal views, respectively **54, 55**. Aponedyopus latilobatus sp. n., ♂ holotype, right gonopod and telopodite tip, mesal view, respectively **56, 57**, right gonopod, lateral view **58**. Scale bar = 0.5 mm.

#### Diagnosis:

Apparently being the most similar to Aponedyopus similis sp. n., it differs in the texture of the tegument (mostly rugulose in Aponedyopus latilobatus sp. n.) and, especially, in certain details of gonopod structure: lobe **l** is neither so wide nor membranous, the terminal branches are subequal in length, and the solenomere is supplied with a far more evident terminal lobe (see also Key below).

#### Description:

Length 15 mm (♂, n=1) and 18 mm (♀, n =3); width of pro- and metazona 10 ca 1.8 and 2.0 (♂) or 1.9–2.0 and 2.0–2.2 mm (♀), respectively.

Coloration in alcohol entirely light brown to brown (Figs 32–35); antennae light brown, growing increasingly blackish distally, but tip pallid; pattern much clearer in ♀, much like in Aponedyopus similis sp. n.: a light brown,wide, axial stripe from anterior edge of collum to end of epiproct; paraterga and sternites contrastingly lighter brown; legs pallid to yellow; axial line wanting.

Postcollum constriction clear (♂) or faint (♀), segment 4 < 3 < 2 < collum = segments 5–16 < head (♂), or collum = segments 2–4 < head = segments 5–18 (♀), thereafter body gradually and gently tapering both in width and height towards telson. Antennae (Fig. 33) medium-sized (♂) to short (♀), slender, reaching behind stricture of tergite 3 dorsally (♂), or end of collum to posterior edge of segment 2 (♀). Paraterga (Figs 34 & 35) as in Aponedyopus similis sp. n., but sometimes not or nearly not delimited by a ventral sulcus (♀). Surface transversely rugulose on metaterga 2 close to paraterga, sparsely longitudinally rugulose in places on post-sulcus halves of metaterga. Pleurosternal carinae (Fig. 33) present only on segments 2 and 3. Tergal setae almost fully abraded, 3+3 retained only at anterrior edge of collum; pattern untraceable. Epiproct (Figs 36 & 37) same as in Aponedyopus similis sp. n., but tip either slightly concave or subtruncate.

Sterna sparsely setose; lamina (Fig. 39) between ♂ coxae 4 evidently emarginate and setose; ♂ segment 7 with a pair of prominent, ventral, sternal cones (= spiracles) flanking gonopod aperture. Legs short and slender, shorter than to almost as long as midbody height; tarsal brushes present from legpair 1 to anterior legs of segment 10; coxa 2 with a small apical process supporting a gonopore.

Gonopod (Figs 46–49, 56 & 57) with **b** more like in Aponedyopus montanus, but **l** especially indistinct, and distal part of solenophore (**sph**), albeit also deeply bifid, having both terminal branches of subequal length, as well as a far more evident terminal lobe on solenomere (**sl**) not crossing a simple end of **sph**.

#### Distribution:

This seems to be a very local high-montane species in central Taiwan (Map).

## Key to Aponedyopus species (based on adult males):

**Table:** 

1	Midbody legs about twice as long as body height. Gonopod with terminal part of solenophore divided into two slender branches (**x** and **y**, Figs 40–43, 50–53)	Aponedyopus montanus
–	Midbody legs only up to 1.2 times as long as body height. Gonopod with terminal part of solenophore divided into wide branches	2
2	Terminal branches of solenophore differing in length and crossing each other, branch **y** with a rather inconspicuous terminal lobe (Figs 44, 45, 54 & 55)	Aponedyopus similis sp. n.
–	Terminal branches of solenophore subequal in length and not crossing each other, branch **y** with a highly inconspicuous terminal lobe (Figs 46–49, 56–58)	Aponedyopus latilobatus sp. n.

## Discussion

Aponedyopus seems to be a small genus endemic to Taiwan. Based on available information, among its three constituent species two are pretty local in distribution, Aponedyopus latilobatus sp. n. and Aponedyopus similis sp. n., each restricted to the northern or central, mostly montane parts of the island, respectively (Map). In contrast, Aponedyopus montanus appears to be extremely widespread, living at various elevations in all parts of Taiwan, including the small islet of Lanyu off the southeastern coast of Taiwan. Whether this species could have been introduced to, and originally described from, Japan, remains open to question. There is only a single example of a basically Taiwanese paradoxosomatid to have become successfully established at least in southern Japan: Chamberlinius hualienensis Wang, 1956 in Kyushu Island and the Ryukyus (Higa and Kishimoto 1986, 1989; Yamaguchi et al. 2000; Nijima and Arimura 2002).

**Map.**
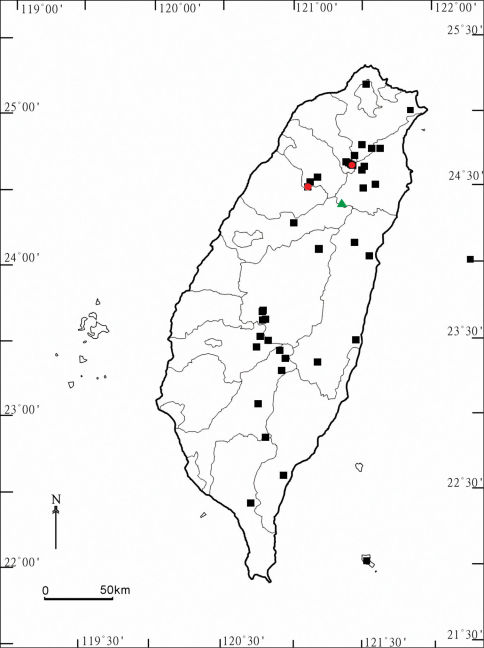
Distribution of Aponedyopus species in Taiwan. Aponedyopus montanus Verhoeff, 1939: filled black squares; Aponedyopus similis sp. n.: filled red circles; Aponedyopus latilobatus sp. n.: filled green triangle.

The distribution of Aponedyopus species in Taiwan shows allopatry. Syntopic occurrences are nearly missing, a feature already reported, e.g., for the paradoxosomatid genus Anoplodesmus (Chen et al. 2010), but contrasting with several other adequately known diplopod groups in Taiwan, in which 2–3 congeners are often capable of sharing the same habitat (Chen et al. 2006; Golovatch et al. 2010; Mikhaljova et al. 2010).

Concerning the tribal position of Aponedyopus, it has long been placed in the tribe Tonkinosomatini (Jeekel 1968; Hoffman 1980). However, we rather think that the few basically East to Southeast Asian paradoxosomatid genera forming the tribe Chamberlinini are actually the closest to Aponedyopus. Yet, no formal transfer is advanced here prior to a revision of the type genus Chamberlinius Wang, 1956 (Chen et al., in preparation).

## Supplementary Material

XML Treatment for Aponedyopus

XML Treatment for Aponedyopus montanus

XML Treatment for Aponedyopus similis

XML Treatment for Aponedyopus latilobatus

## References

[B1] Chen CC Golovatch SI Chang HW (2006) The millipede tribe Nedyopodini, with special reference to the fauna of Taiwan (Diplopoda: Polydesmida: Paradoxosomatidae). Journal of Natural History 39 (47):3997-4030

[B2] Chen CC Golovatch SI Mikhaljova EV Chang HW (2010) The millipede genus *Anoplodesmus* Pocock, 1895, recorded in Taiwan for the first time, with descriptions of two new species (Diplopoda: Polydesmida: Paradoxosomatidae: Sulciferini). Zootaxa 2399:20-30

[B3] Golovatch SI Mikhaljova EV Chang HW (2010) Pill-millipedes (Glomerida, Diplopoda) in Taiwan. Zootaxa 2477:1-20

[B4] Higa Y Kishimoto T (1986) Unusual outbreak and control of millipedes, *Chamberlinius haulienensis* Wang in Okinawa. Annual Report of Okinawa Prefectural Institute of Public Health 20: 62–72 (In Japanese)

[B5] Higa Y Kishimoto T (1989) Expansion of distribution area of millipede, *Chamberlinius haulienensis* Wang, in Okinawa. Annual Report of Okinawa Prefectural Institute of Public Health 23: 72–76 (In Japanese)

[B6] Hoffman RL (1980) Classification of the Diplopoda. Muséum d’histoire naturelle, Genève, 237 pp. [for 1979]

[B7] Jeekel CAW (1968) On the classification and geographical distribution of the family Paradoxosomatidae (Diplopoda, Polydesmida). Academisch Proefschrift, Rotterdam, 162 pp.

[B8] Korsós Z (2004) Checklist and bibliography of millipedes (Diplopoda) of Taiwan. Collection and Research 17:11-32

[B9] Mikhaljova EV Golovatch SI Chang HW (2010) The millipede family Diplomaragnidae in Taiwan, with descriptions of nine new species (Diplopoda, Chordeumatida). Zootaxa 2615:23-46

[B10] Miyosi Y (1959) Über japanische Diplopoden. Arachnological Society of East Asia, Osaka, 223 pp, plates 1–19. (In Japanese)

[B11] Murakami Y (1993) Diplopoda, Pauropoda, Symphyla. A list of Japanese species. Invertebrates 1. Shizen-Kankyô-Kenkyû Center. Environmental Agency Japan, Tokyo, 95–106 (In Japanese)

[B12] Nijima K Arimura T (2002) Obstruction of trains by the outbreaks a millipede *Chamberlinius hualienensis* Wang (Diplopoda: Polydesmida). Edaphologia 69: 47–49 (In Japanese)

[B13] Shinohara K Tanabe T (1999) Diplopoda. In: Aoki J (Ed) Pictorial Keys to Soil Animals of Japan. Tokai University Press, Tokyo, 647–683 (In Japanese).

[B14] Takakuwa Y (1942) Einige neue Arten von Diplopoda aus Nippon. Zoological Magazine, 54:237-239

[B15] Takakuwa Y (1954) Diplopoden aus Japan und ihr angrenzenden Gebieten. Japan Society for Promotion of Science, Tokyo, 241 pp. (In Japanese)

[B16] Tanabe T (2001) The biology of centipedes and millipedes. Tokai University Press, Tokyo, i-xi + 178 pp.

[B17] Verhoeff KW (1939) Zur Kenntnis ostasiatischer Diplopoden. III. Zoologischer Anzeiger 127:115-125

[B18] Wang D Mauriès JP (1996) Review and perspective of study on myriapodology of China. In: Geoffroy JJ, Mauriès JP, Nguyen Duy-Jacquemin M (Eds) Acta Myriapodologica. Mémoires du Muséum National d’Histoire Naturelle 169:81-99

[B19] Wang YHM (1957a) Serica 1g: Records of Myriapods on Taiwan Islands (4) Six new polydesmids. Quarterly Journal of the Taiwan Museum 10:103-111

[B20] Wang YHM (1957b) Serica 1h: Records of myriapods on Taiwan Islands (5) with description of three new species. Quarterly Journal of the Taiwan Museum 10:113-116

[B21] Wang YHM (1958) Serica 1i, On Diplopoda from Taiwan with a new strongylosomids. Quarterly Journal of the Taiwan Museum 11:340-344

[B22] Wang YHM (1963a) Serica 1Q: Millipedes and centipedes of Quemoy, Fukien Province and Taiwan Island, Botel Tobago (Lan Yu), Taiwan Province and of Singapore. Quarterly Journal of the Taiwan Museum 16:89-96

[B23] Wang YHM (1963b) On millipedes and centipedes from Taiwan, China. Proceedings of the XVIth International Congress of Zoology, Washington D.C., 1963 1: 285, 288–291

[B24] Wang YHM (1964) Serica 1 op: Wallacea and insular fauna of millipedes. Quarterly Journal of Taiwan Museum 17:67-76

[B25] Yamaguchi T Izumi S Takemura K Torigoe H Matunaga T. Nagata K (2000) Annual occurrence of *Chamberlinius haulienensis* Wang on Amami-Oshima Island and possible chemicals for controlling it. Proceedings of the Association for Plant Protection of Kyushu 46: 118–122 (In Japanese)

